# *Bordetella Pertussis* Adenylate Cyclase Toxin Does Not Possess a Phospholipase A Activity; Serine 606 and Aspartate 1079 Residues Are Not Involved in Target Cell Delivery of the Adenylyl Cyclase Enzyme Domain

**DOI:** 10.3390/toxins10060245

**Published:** 2018-06-16

**Authors:** Ladislav Bumba, Jiri Masin, Adriana Osickova, Radim Osicka, Peter Sebo

**Affiliations:** Institute of Microbiology of the CAS, v.v.i., 142 20 Prague, Czech Republic; bumba@biomed.cas.cz (L.B.); masin@biomed.cas.cz (J.M.); osickova@biomed.cas.cz (A.O.); osicka@biomed.cas.cz (R.O.)

**Keywords:** adenylate cyclase toxin, phospholipase A activity, AC domain translocation

## Abstract

The adenylate cyclase toxin-hemolysin (CyaA, ACT, or AC-Hly) plays a crucial role in virulence and airway colonization capacity of the whooping cough agent *Bordetella pertussis*. The toxin penetrates target cell membranes and exhibits three distinct biological activities. A population of CyaA conformers forms small cation-selective pores that permeabilize the cell membrane for potassium efflux, which can provoke colloid-osmotic (oncotic) cell lysis. The other two activities are due to CyaA conformers that transiently form calcium influx conduits in the target cell membrane and translocate the adenylate cyclase (AC) enzyme into cytosol of cells. A fourth putative biological activity has recently been reported; an intrinsic phospholipase A (PLA) activity was claimed to be associated with the CyaA polypeptide and be involved in the mechanism of translocation of the AC enzyme polypeptide across cell membrane lipid bilayer. However, the conclusions drawn by the authors contradicted their own results and we show them to be erroneous. We demonstrate that highly purified CyaA is devoid of any detectable phospholipase A1 activity and that contrary to the published claims, the two putative conserved phospholipase A catalytic residues, namely the Ser606 and Asp1079 residues, are not involved in the process of membrane translocation of the AC domain of CyaA across target membranes.

## 1. Introduction

The adenylate cyclase toxin-hemolysin (ACT, AC-Hly, or CyaA) is a key virulence factor of *Bordetellae* that are pathogenic to mammals [1]. The toxin belongs to the Repeats-in-ToXin (RTX) family of proteins that are excreted by Gram-negative bacteria through the Type I secretion system [2,3,4]. CyaA is a multifunctional protein (1706 residues) that consists of an N-terminal enzymatic adenylate cyclase (AC) domain of 384 residues that is fused to a pore-forming RTX hemolysin moiety (Hly) of 1322 residues by an AC-to-Hly-linker segment [5,6,7].

The Hly moiety of CyaA includes a hydrophobic pore-forming domain, a fatty acyl-modified domain, an RTX calcium-binding domain, and a C-terminal secretion signal [8]. The binding of about 40 calcium ions by the glycine- and aspartate-rich repeats of the RTX domain, which induces folding of the toxin molecule and the covalent fatty-acylation of the ε-amino groups of lysine residues 860 and 983 by the acyltransferase CyaC [9], are both essential prerequisites for the CyaA toxin molecule to interact with its receptor on myeloid phagocytic cells, the heterodimeric α_M_β_2_ integrin CD11b/CD18, known as the complement receptor 3 (CR3), or Mac-1 [10,11]. Upon interaction with N-linked oligosaccharides [12,13], the CyaA recognizes a specific segment of the CD11b subunit of CR3 [11], which positions it for efficient insertion into the phagocyte membrane and enables the toxin molecule to directly deliver its N-terminal AC domain across the cytoplasmic membrane into the cytosol of the cell, without the need for receptor-mediated endocytic uptake of the toxin [10,14,15,16,17,18,19,20].

The Hly part of CyaA can also mediate low efficacy binding to cells that lack the CR3 receptor, presumably through an unspecific low affinity interaction with cellular glycoproteins and glycolipids, which enables the low-level penetration of CyaA across the membranes of a number of cells types, such as epithelial cells or even erythrocytes. Translocation of the AC domain of CyaA into such cells then generates a measurable increase in cytosolic cAMP concentrations sufficient for interference with cellular signaling [21,22]. Membrane-inserted CyaA molecules are further able to oligomerize into cation-selective hemolytic pores that permeabilize the cell membrane and enable the efflux of potassium ions from cells [23,24,25,26]. This can eventually cause colloid-osmotic (oncotic) lysis of cells [21].

The mechanistic details of AC domain translocation across the lipid bilayer of the cellular plasma membrane remain poorly understood. Earlier, we showed that a membrane translocation intermediate of the AC domain itself participates in the formation of a novel type of conduit that mediates the influx of extracellular calcium ions across the plasma membrane of monocytic cells [27]. Calcium-dependent activation of calpain, cleavage of talin, and recruitment of the CyaA-CR3 complex into the cholesterol-rich lipid membrane microdomains (lipid rafts) then follows, where the raft lipid packing enables completion of the AC domain translocation across the cell membrane bilayer [28]. The AC domain translocates across the cytoplasmic membrane of cells with a very short half-time; this process appears to be driven by membrane potential [29,30]. The AC membrane translocation further requires an overall positive net charge of the AC domain [31]. An “AC-to-Hly-linking” segment of CyaA was identified and its arginine residues appear to be involved in the disruption of the membrane bilayer, which enables insertion and translocation of the AC domain of CyaA across the plasma membrane of cells [5,6,7]. Furthermore, the structural integrity of the putative transmembrane helices I, III, and IV of the hydrophobic domain was shown to be essential for AC domain translocation across the plasma membrane of both CD11b^+^ and CD11b^−^ cells [32,33,34,35].

Gonzáles-Bullón et al. [36] reported that in addition to pore-forming, calcium-conducting, and AC-translocating activities, the CyaA polypeptide possesses an intrinsic calcium-dependent phospholipase A (PLA) activity. This was proposed to be directly involved in the translocation of the AC domain of CyaA across PLA-formed toroidal pores within the lipid bilayer of the cellular membrane. However, the conclusions reached by the authors directly contradicted the data presented in their report, and given the absence of appropriate controls, a number of simpler explanations of the reported data remained plausible (see our Letter in *Proc. Natl. Acad. Sci. USA*. [37]). Although Gonzáles-Bullón et al. claimed to have discovered a new enzymatic activity of CyaA, the enzyme kinetics of the putative PLA activity of CyaA were not shown. Moreover, the authors claimed that the Ser606 and Asp1079 residues of CyaA are directly involved as catalytic residues in the intrinsic PLA catalytic activity of CyaA and were thus importantly involved in AC domain translocation across the lipid bilayer of the target cell membrane by the toxin polypeptide. The discovery of an intrinsic PLA activity of CyaA in addition to its AC enzyme and pore-forming cytotoxic activities would, indeed, be of high relevance for our understanding of the mechanism by which CyaA translocates its AC domain across target cell membrane. Therefore, we decided to verify if the CyaA polypeptide possesses an intrinsic PLA enzyme activity. The results reported here clearly show that a highly purified and fully biologically active CyaA toxin is devoid of any detectable intrinsic PLA-1 enzyme activity.

## 2. Results and Discussion

**Figure 1:**
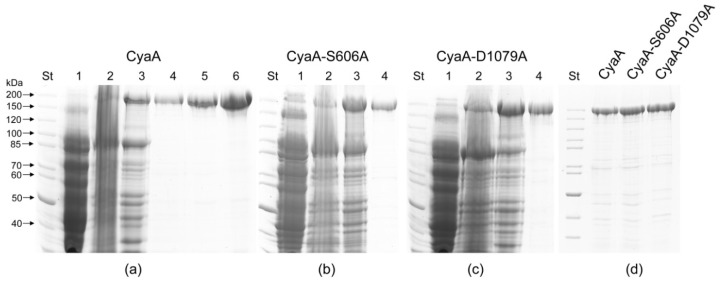
SDS-PAGE analysis of the CyaC-acylated CyaA protein variants. (**a**) Intact CyaA and its mutant variants (**b**) CyaA-S606A and (**c**) CyaA-D1079A were produced in appropriately transformed *E. coli* BL-21 cells. The individual fractions included: (1) the cytosolic extract of CyaA-producing *E. coli* cells disrupted by sonication, (cell lysate); (2) the bacterial membrane fraction recovered by washing of inclusion bodies in 4 M urea buffer (membranes); (3) the crude preparation of 8 M urea-solubilized CyaA-containing inclusion bodies (urea extract); (4) CyaA purified by anion-exchange chromatography under denaturing conditions in 8 M urea buffer on DEAE-Sepharose column (DEAE); (5) the CyaA re-purified after the DEAE column by affinity chromatography on Calmodulin Agarose (DEAE + CaM); and (6) CyaA finally purified by hydrophobic chromatography on Phenyl-Sepharose (DEAE + CaM + Phenyl). (**d**) Side-by-side comparison of electrophoretic mobility of intact CyaA and of its S606A and D1079A variants. St: molecular weight standards.

To test if purified CyaA exhibits a PLA activity, we produced the recombinant CyaC-activated CyaA in *E. coli* BL-21 cells and purified the toxin close to homogeneity from eight molar urea extracts of washed inclusion bodies. The toxin purification fractions included (1) the CyaA purified by anion-exchange chromatography under denaturing conditions in eight molar urea buffer on a Diethylaminoethyl-Sepharose column (DEAE), (2) the CyaA re-purified after the DEAE column by affinity chromatography on Calmodulin Agarose (DEAE + CaM), and (3) a CyaA finally purified on a third column by hydrophobic chromatography on Phenyl-Sepharose (DEAE + CaM + Phenyl). As shown in Figure 1a, the homogeneity of the CyaA preparations in terms of protein band pattern on intentionally overloaded 7.5% SDS-PAGE gel increased significantly between the membrane and urea extract fractions. The homogeneity further improved modestly after single, double, or triple chromatographic purification.

**Figure 2:**
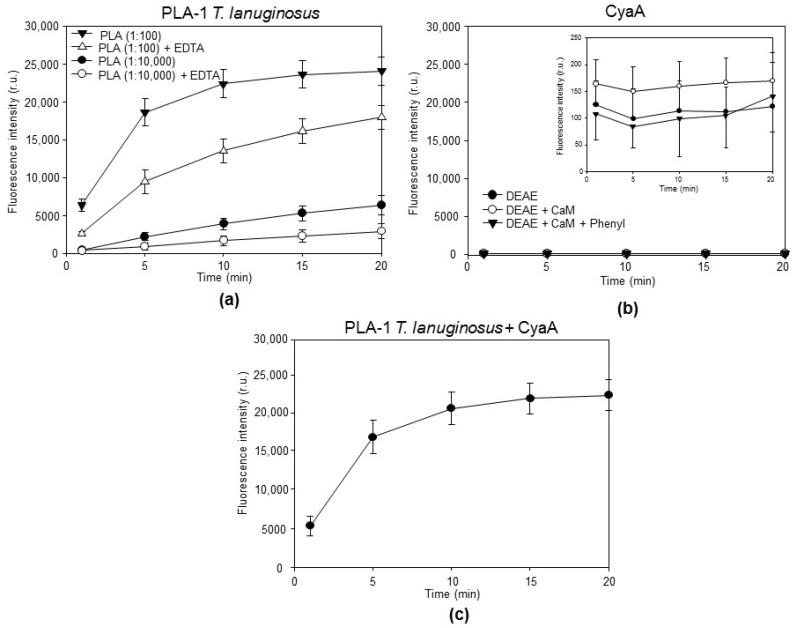
The purified CyaA toxin is devoid of any detectable phospholipase A1 activity. (**a**) The stock solution of phospholipase A1 from *Thermomyces lanuginosus* was diluted 1:100 or 1:10,000 in 10 mM Tris-HCl (pH 7.4), 100 mM NaCl, and 10 mM CaCl_2_ (or 20 mM EDTA), and incubated with 500 nM PED-A1, a selective PLA-1 fluorogenic substrate, before the fluorescence emission at 530 nm (excitation at 488 nm) was recorded under a continuous kinetic readout in the microtiter plate. Fluorescence intensities of the cleaved fluorogenic substrate were subtracted from the background and averaged. The values represent the average ± standard deviations derived from three independent experiments performed in triplicate (*n* = 9). (**b**) Individual purified CyaA fractions were diluted in buffer to provide a final AC enzyme activity of 1 U/mL. Incubation with 500 nM PED-A1 occurred before fluorescence emission at 530 nm was recorded at indicated time points of *t* = 1, 5, 10, 15, and 20 min. The values represent the average ± standard deviations derived from three independent experiments performed in triplicate with two independent CyaA preparations (*n* = 9). (**c**) The 500 nM PED-A1 substrate was mixed with highly purified CyaA (DEAE + CaM + Phenyl, 1 U/mL) and 1:100 diluted phospholipase A1 from *Thermomyces lanuginosus* was added. Fluorescence emission at 530 nm was recorded.

Individual fractions from different CyaA purification steps were diluted in 150 mM NaCl, 20 mM Tris-HCl, and 10 mM CaCl_2_, in pH 8.0 buffer to provide a final AC enzyme activity of 1 U/mL (~2.5 µg/mL of CyaA) and the toxin fractions were incubated with 500 nM PED-A1 substrate. Cleavage of the sn–1 ester bond of the PED-A1 substrate by the phospholipase A1 activity would induce the release of the corresponding fatty acid and can thus be measured as an increase in the fluorescence intensity at 530 nm. A well-detectable activity of a diluted preparation of a positive control enzyme, the calcium-dependent phospholipase A1 from *Thermomyces lanuginosus,* was observed (Figure 2a). In contrast, no detectable increase in the fluorescence intensity at 530 nm was observed in the sole presence of 2.5 µg/mL of any of the purified CyaA proteins (Figure 2b). To confirm the findings that the purified CyaA preparations did not contain components that could block PED-A1 substrate cleavage, the reaction mixtures containing 500 nM PED-A1 substrate and 2.5 µg/mL of highly purified intact CyaA (DEAE+CaM+Phenyl fraction) were spiked by the phospholipase A1 enzyme from *T. lanuginosus* (100× diluted stock). As shown in Figure 2c, the PLA-1 enzyme of *T. lanuginosus* then cleaved the PED-A1 substrate with the same efficacy as in the absence of added CyaA (Figure 2a). These data show that the purified intact CyaA toxin itself does not possess any detectable intrinsic PLA-1 enzyme activity.

Gonzáles-Bullón et al. [36] further proposed that the serine residue 606 (S606) and the aspartate residue 1079 (D1079) of CyaA were involved in the formation of a catalytic site of a patatin family-like phospholipase A enzyme within the structure of the CyaA protein. To corroborate this, the authors replaced the S606 and D1079 residues of CyaA by alanine residues and reported that a preparation of the S606A mutant CyaA exhibited an importantly reduced PLA-1 and PLA-2 phospholipase activity. Compared to intact CyaA, the CyaA-S606A toxin then exhibited a modestly reduced (~50%) cytotoxic activity on J774A.1 cells. In contrast, the CyaA-D1079A mutant was found to be inactive as a toxin, despite its rather high PLA activity (Figures 4, 6, and 8 and Supplementary Table S1 in Gonzáles-Bullón et al. [36]). Notwithstanding the obvious contradiction of the low PLA activity of CyaA-S606A being accompanied by a rather high specific cytotoxic activity, whereas high PLA activity accompanied the complete loss of cytotoxic activity of CyaA-D1079A, the authors concluded that the PLA activity was intrinsic to CyaA and was essential for the capacity of the toxin to deliver the AC enzyme domain into the cell cytosol for exerting its cytotoxic action.

**Figure 3:**
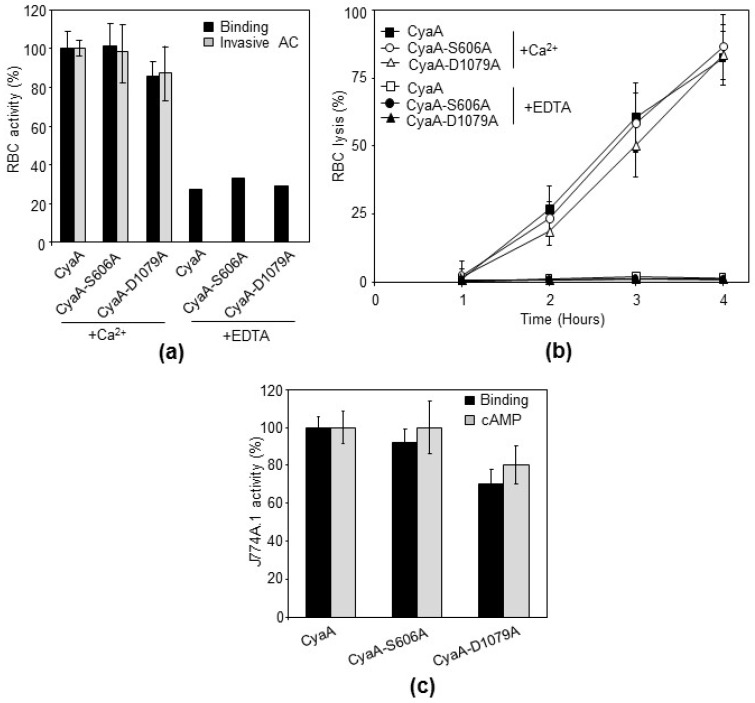
CyaA-S606A and CyaA-D1079A toxins exhibit an almost identical specific cytotoxic activity against sheep erythrocytes and mouse macrophages J774A.1 as intact CyaA. Proteins were expressed in *E. coli* BL-21 cells and purified from urea extracts on DEAE Sepharose. (**a**) Sheep erythrocytes (5 × 10^8^ cells/mL) were incubated at 37 °C in the presence of 2 mM calcium (+Ca^2+^) or 5 mM EDTA (+EDTA) with 1 µg/mL (~0.4 U/mL) of the purified CyaA proteins and after 30 min. Aliquots were used for determination of the cell-associated AC activity (binding) and of the AC activity internalized into erythrocytes and protected against digestion by externally added trypsin (invasive AC). Activities are expressed as percentages of intact CyaA activity and represent average values ± standard deviations from at least three independent determinations performed in duplicate with two different toxin preparations (*n* = 6–8). (**b**) Sheep erythrocytes (5 × 10^8^ cells/mL) in Tris 50 mM, NaCl 150 mM, CaCl_2_ 2 mM, pH 7.4 buffer (TNC) were incubated at 37 °C with intact CyaA or with its mutant variants (10 µg/mL). Hemolytic activity was measured as the amount of released hemoglobin by photometric determination (A_541_), (*n* = 3). (**c**) Binding of intact CyaA or its mutant variants to J774A.1 cells (1 × 10^6^) was determined as the amount of total cell-associated AC enzyme activity upon incubation of cells with 1 µg/mL (~0.4 U/mL) of the protein for 30 min at 4 °C. cAMP intoxication was assessed by determining the intracellular concentration of cAMP generated in cells after 30 min of incubation of J774A.1 cells (2 × 10^5^) with four different toxin concentrations from within the linear range of the dose-response curve (100, 50, 25, and 12.5 ng/mL, i.e., ~40, 20, 10, 5 mU/mL, respectively). Activities are expressed as percentages of intact CyaA activity and represent average values ± standard deviations from at least three independent determinations performed in duplicate with two different toxin preparations (*n* = 6–8).

To address this issue, we constructed the same mutated variants of CyaA and verified the introduction of the desired residue substitutions and the absence of unrelated mutations by DNA sequencing of the respective plasmid constructs. The mutant CyaA-S606A and CyaA-D1079A toxin variants were then purified close to homogeneity (Figure 1b,c) and exhibited the same mobility on an 7.5% SDS-PAGE gel (Figure 1d). The capacity of the purified proteins to deliver the AC domain into the cytosol of target cells and elevate the cytosolic concentrations of cAMP was then compared to that of intact purified CyaA. The three purified CyaA protein variants were first tested for the capacity to deliver the AC enzyme across the sheep erythrocyte membrane into a trypsin-inaccessible cytosolic compartment (cell-invasive AC activity). As documented in Figure 3a, the CyaA-D1079A protein exhibited about 85% of the specific cell-binding activity of the intact CyaA and the CyaA-S606A variant binding was indistinguishable from that of intact CyaA.

All three examined toxin variants delivered the AC enzyme into the cytosol of the erythrocytes, where the enzyme was protected against digestion by the externally added trypsin. Moreover, all three toxin variants exhibited about the same specific capacity to form hemolytic pores within the erythrocyte membrane and provoked the colloid-osmotic lysis of erythrocytes (Figure 3b). Therefore, we next examined the capacity of the CyaA constructs to bind the CR3 receptor on phagocytes and to penetrate mouse macrophage J774A.1 cells [10,11]. When measured as the level of intracellularly accumulated cAMP in cells exposed to these toxins, the intact CyaA and the two mutant proteins exhibited an almost identical specific cytotoxic activity. As documented in Figure 3c, the CyaA-D1079A protein exhibited about 70% of the specific cell-binding capacity and about 80% of the AC domain translocation capacity (cAMP intoxication) of the intact CyaA on a per AC enzyme unit basis, whereas the cell-binding and AC domain translocating capacities of the CyaA-S606A protein were indistinguishable from those of the intact CyaA. The observation that the CyaA-S606A toxin was fully active in AC domain delivery into cell cytosol aligns well with our previous report that the insertion of a ValTyrThr tripeptide between the Ser606 and Gly607 residues of CyaA had no effect whatsoever on the capacity of the mutated toxin to penetrate the sheep erythrocyte membrane and deliver its AC domain into the cytosol of these cells [38]. We further showed in the same report that the insertion of a larger VYSIINFEKLGYT peptide between the Ser606 and Gly607 residues of CyaA only decreased the specific membrane insertion capacity of the mutated CyaA, whereas the ability of the already membrane-inserted mutant CyaA protein to translocate the AC domain (enzyme translocation efficacy) was intact. It is highly unlikely that VYSIINFEKLGYT or VYT peptide insertion next to the putative S606 catalytic residue of the proposed PLA enzyme domain of CyaA would not disrupt its putative PLA enzyme catalytic site. The evidence presented here, that ACT-S606A is fully active as an AC toxin, similar to the CyaA-S606VYT607G or CyaA-S606VYSIINFEKLGYT608G proteins [38], makes it extremely unlikely that even if CyaA possessed an intrinsic PLA activity involving Ser606 as catalytic residue, that this would play any role in the capacity of the CyaA toxin to translocate its AC enzyme domain across target membrane lipid bilayer.

The CyaA-S606A toxin of Gonzáles-Bullón et al. [36] did exhibit a modestly reduced AC translocation capacity on J774A.1 cells (cAMP intoxication), ranging around 50% of the specific capacity of the intact CyaA (Figure 6a in Gonzáles-Bullón et al. [36]). This reduction was most likely artefactual and was probably due to inappropriate assay conditions, since incubation of J774A.1 cells with the CyaA concentrations used by the authors, higher than 1 nM, leads to rapid exhaustion of cytosolic ATP within minutes, which causes the cAMP accumulation assays to be very unreliable and imprecise [27,33,39]. The analysis of intracellular cAMP content at ATP-depleting conditions, such as using a 5 nM CyaA concentration for 30 min, necessarily yields erroneous results.

Gonzáles-Bullón et al. [36] used a degraded or truncated CyaA-D1079A protein, which migrated faster on SDS-PAGE gels than intact CyaA (see their Supplementary Figure S6 and S8 [36]), which was thus devoid of CyaA toxin activity. In contrast, our results showed that the non-degraded full-length CyaA-D1079A protein exhibits the same mobility on SDS-PAGE gels as the intact CyaA (Figure 1d) and is highly active as a cell-penetrating AC toxin. Depending on the used target cell type, the D1079A substitution decreased the specific membrane insertion capacity of CyaA by 15 to 30%, as compared with intact toxin (Figure 3a,c), whereas the capacity of the already membrane-bound CyaA-D1079A protein to translocate the AC domain (translocation efficacy) remained intact. The D1079A substitution in the RTX block I of CyaA may slightly affect the calcium binding properties and thus the calcium-dependent folding of the RTX domain that is required for toxin binding to the CR3 receptor and/or for membrane insertion of CyaA [2]. The CyaA-D1079A protein used by Gonzáles-Bullón et al. [36] exhibited full AC enzyme activity, which is located in the N-terminal AC domain (Figure 6b, in Gonzáles-Bullón et al. [36]). However, it migrated faster on SDS-PAGE gels than intact CyaA (see their Supplementary Figures S6 and S8 [36]). The used CyaA-D1079A was therefore truncated in the C-terminal RTX domain, which offers an obvious explanation for the loss of the cell-invasive AC and cytotoxic activity of CyaA-D1079A protein observed by the authors [36]. Indeed, the structural integrity of the RTX domain was repeatedly shown to be essential for the capacity of CyaA to bind calcium ions and undergo the conformational change that is essential for the cell-invasive activity of CyaA on cells lacking the CR3 receptor [15,18,40]. Moreover, the structural integrity of the RTX domain is also essential for the capacity of CyaA to bind target cells through the CR3 receptor molecule to penetrate the phagocyte membrane and deliver across the AC domain [2,41]. The reported comparison of the membrane-binding activities of CyaA-D1079A only on liposomes (Supplementary Figure S8 in Gonzáles-Bullón et al. [36]) is then irrelevant with respect to the biological activities of the CyaA protein, because the presence of the calcium-binding RTX domain was previously shown to be dispensable for insertion of CyaA fragments into the naked lipid bilayer membranes of liposomes [42].

In conclusion, we regret to summarize our above reported results by concluding that the claimed varying levels of the PLA activities in the CyaA and CyaA-S606A and CyaA-D1079D preparations used in the work of Gonzáles-Bullón et al. [36] were likely due to contamination of their toxin samples by some residual amounts of a calcium-dependent PLA enzyme produced by *E. coli* K12 cells. Alternatively, a problem with the performance of the PLA assays and reporting of misleading ratios of fluorescence values at a single time point, in the absence of time-dependent PLA enzyme kinetics, remains a possibility as well. Furthermore, as shown here, even if this was not the case and the S606A and D1079A residues are really a part of a PLA catalytic motif in the CyaA protein, this hypothetical intrinsic PLA activity of CyaA would not play any role whatsoever in the cytotoxic activity of CyaA. This is because the CyaA-S606A and CyaA-D1079A proteins were highly active as AC toxins, both on cells having the CR3 integrin, performing CyaA binding on J774A.1 macrophage cells, as well as on erythrocytes that lack the integrin molecule and bind CyaA at much reduced levels. We thus conclude that highly purified CyaA protein is devoid of any intrinsic phospholipase A activity that would play any role in the capacity of the toxin to penetrate target cell plasma membranes.

## 3. Materials and Methods

### 3.1. Construction, Production, and Purification of *CyaA* Proteins

The pT7CACT1 plasmid was used for co-expression of *cyaC* and *cyaA* genes, allowing for the production of the recombinant CyaC-activated CyaA proteins in *Escherichia coli* [38]. Oligonucleotide-directed polymerase chain reaction (PCR) mutagenesis was used to construct pT7CACT1-derived plasmids for expression of the CyaA-S606A and CyaA-D1079A mutant variants. Intact CyaA and its mutant variants were produced in the *E. coli* strain BL21 (Novagen, Birmingham, UK) carrying the plasmid pMM100 (encoding LacI and tetracycline resistance), which was transformed with appropriate pT7CACT1-derived constructs. The cells were grown at 37 °C in MDO medium (yeast extract, 20 g/L; glycerol, 20 g/L; KH_2_PO_4_, 1 g/L; K_2_HPO_4_, 3 g/L; NH_4_Cl, 2 g/L; Na_2_SO_4_, 0.5 g/L; and thiamine hydrochloride, 0.01 g/L) supplemented with 150 μg/mL of ampicillin and 12.5 μg/mL of tetracycline, induced at OD_600_ = 0.6 with 1 mM isopropyl 1-thio-*β*-d-galactopyranoside (IPTG), and grown for an additional 4 h. Then the cells were collected, disrupted by ultrasound, and the insoluble cell membrane fraction, covering the tightly packed whitish inclusion body pellet, was first mechanically removed using a spatula and the rest of the fluffy brownish membrane pellet was resuspended in 4 M urea in 50 mM Tris-HCl (pH 8.0) and removed by pipetting. The rinsed pellets of the insoluble inclusion bodies, remaining attached to centrifugation tube wall, were then solubilized and extracted with 8 M urea in 50 mM Tris-HCl (pH 8.0). The CyaA proteins were purified from 8 M urea extracts by ion-exchange chromatography on DEAE-Sepharose, by affinity chromatography on Calmodulin-Agarose and by hydrophobic chromatography on Phenyl-Sepharose, as previously described [5,43]. Concentrations of the purified CyaA proteins were determined by the Bradford assay (Bio-Rad, Hercules, CA, USA). The integrity of all proteins was systematically confirmed by SDS-PAGE.

### 3.2. Cell Binding, Cell Invasive, and Hemolytic Activities on Sheep Erythrocytes

Adenylate cyclase enzymatic activities were measured in the presence of 1 µM calmodulin (CaM) as previously described [44]. One unit (1 U) of adenylate cyclase activity corresponds to 1 µmol of cAMP formed per min at 30 °C, pH 8.0. Hemolytic activities were measured in TNC (50 mM Tris-HCl at pH 7.4, 150 mM NaCl, and 2 mM CaCl_2_) or TNE buffer (50 mM Tris-HCl at pH 7.4, 150 mM NaCl, and 5 mM EDTA) by determining the hemoglobin release in time upon CyaA incubation (10 µg/mL) with washed sheep erythrocytes (5 × 10^8^/mL in TNC or TNE), as previously described [21]. Erythrocyte binding and cell-invasive AC activities were determined as described in detail previously [21,40]. Briefly, sheep erythrocytes (5 × 10^8^ cells/mL) were incubated with CyaA (1 µg/mL) at 37 °C in TNC/TNE buffer. After 30 min, cell suspensions were washed in TNC buffer to remove unbound CyaA and divided in two aliquots. The first aliquot was directly used to determine the amount of cell-associated AC activity (membrane-bound CyaA). The second aliquot was treated with 20 μg/mL of trypsin for 15 min at 37 °C to inactivate the extracellular AC toxin, which did not translocate into cells. Soybean trypsin inhibitor (40 μg/mL) was added to the mixture to stop the reaction before the samples were washed in TNE buffer and used to determine the amount of cell-invasive AC activity. Activity of intact CyaA was taken as 100%.

### 3.3. Binding and cAMP Elevation of ACT on J774A.1 Cells

J774A.1 murine monocytes and macrophages (ATCC, number TIB-67) were cultured at 37 °C in a humidified air/carbon dioxide (CO_2_) (19:1) atmosphere in RPMI medium supplemented with 10% (*v*/*v*) heat-inactivated fetal bovine serum, penicillin (100 IU/mL), streptomycin (100 µg/mL), and amphotericin B (250 ng/mL). Prior to assays, RPMI was replaced with Dulbecco’s modified eagle medium (D-MEM) (1.9 mM Ca^2+^) without FCS and the cells were allowed to rest in D-MEM for 1 h at 37 °C in a humidified 5% CO_2_ atmosphere [33]. J774A.1 cells (10^6^) were incubated in D-MEM with 1 µg/mL of CyaA variants for 30 min at 4 °C prior to removal of unbound toxin by three washes in D-MEM. After transfer to a fresh tube, cells were lyzed with 0.1% Triton X-100 for determination of cell-bound AC enzyme activity. For intracellular cAMP assays, 2 × 10^5^ cells were incubated with CyaA (100, 50, 25 and 12.5 ng/mL, i.e., about 40, 20, 10, 5 mU/mL) for 30 min in D-MEM. The reaction was stopped by addition of 0.2% Tween-20 in 100 mM HCl, samples were boiled for 15 min at 100 °C, neutralized by addition of 150 mM unbuffered imidazole, and the concentration of cAMP was determined by a competition immunoassay [31].

### 3.4. Phospholipase A1 Activity Determination

The stock solution of phospholipase A1 from *Thermomyces lanuginosus* (L3295, Sigma, phospholipase A1 activity in KLU/G ≥ 10) was diluted (1:100 and 1:10,000) in 10 mM Tris-HCl (pH 7.4), 100 mM NaCl, and 10 mM CaCl_2_ and incubated with 500 nM selective PLA-1 fluorogenic substrate PED-A1 (*N*-((6-(2,4-DNP)Amino)Hexanoyl)-1-(BODIPY FL C5)-2-Hexyl-Sn-Glycero-3-Phosphoethanolamine, Invitrogen, Carlsbad, CA, USA) before fluorescence emission at 530 nm (after excitation wavelength at 488 nm) was recorded under a continuous kinetic readout in the microtitration plate. Individual fractions from different CyaA preparations were diluted in 10 mM Tris-HCl (pH 7.4), 100 mM NaCl and 10 mM CaCl_2_ buffer to give a final AC enzyme activity of 1 U/mL (~2.5 µg/mL) and incubated with 500 nM PED-A1 before fluorescence emission at 530 nm (after excitation wavelength at 488 nm) was recorded.
